# Serum Osteoprotegerin Is an Independent Marker of Left Ventricular Hypertrophy, Systolic and Diastolic Dysfunction of the Left Ventricle and the Presence of Pericardial Fluid in Chronic Kidney Disease Patients

**DOI:** 10.3390/nu14142893

**Published:** 2022-07-14

**Authors:** Katarzyna Romejko, Aleksandra Rymarz, Katarzyna Szamotulska, Zbigniew Bartoszewicz, Stanisław Niemczyk

**Affiliations:** 1 Department of Internal Diseases, Nephrology and Dialysis, Military Institute of Medicine, 128 Szaserów Street, 04-141 Warsaw, Poland; sniemczyk@wim.mil.pl; 2 Department of Epidemiology and Biostatistics, Institute of Mother and Child, Kasprzaka St., 17a, 01-211 Warsaw, Poland; katarzyna.szamotulska@imid.med.pl; 3 Department of Internal Diseases and Endocrinology, Medical University of Warsaw, 1a Banacha St., 02-097 Warsaw, Poland; zbigniew.bartoszewicz@wum.edu.pl

**Keywords:** osteoprotegerin, chronic kidney disease, heart failure, bioimpedance, left ventricle hypertrophy, ejection fraction, diastolic dysfunction, pericardial fluid

## Abstract

Background: Osteoprotegerin (OPG) is a molecule which belongs to the tumor necrosis factor receptor superfamily. OPG concentration is elevated in patients with left ventricle hypertrophy, heart failure and acute myocardial infarction. OPG concentrations rise in chronic kidney disease (CKD). The aim of this study was to investigate the association between OPG concentrations and cardiovascular complications, such as left ventricle hypertrophy, systolic and diastolic dysfunction of left ventricle and dysfunction of right ventricle in chronic kidney disease patients not treated with dialysis. The relation between OPG and the amount of pericardial fluid was also examined. Methods: One hundred and one men with CKD stage 3–5 not treated with dialysis were included in the study. Overhydration, body fat mass and lean body mass were measured using bioimpedance spectroscopy (BIS). Echocardiography was performed to evaluate the amount of pericardial fluid and to measure the thickness of the interventricular septum (IVS), systolic and diastolic function of left ventricle, as well as systolic function of right ventricle. Results: We observed a significant positive association between OPG and the thickness of the interventricular septum, the size of the left atrium (LA) and the presence of pericardial fluid. A negative relationship was observed between OPG and ejection fraction (EF). Conclusions: Our results suggest that OPG can be an independent marker of left ventricular hypertrophy, systolic and diastolic dysfunction of left ventricle and the presence of pericardial fluid in chronic kidney disease patients.

## 1. Introduction

Osteoprotegerin (OPG) is a glycoprotein which belongs to the tumor necrosis factor (TNF) receptor superfamily. The OPG gene is located on chromosome 8q and consists of five exons. OPG is an inhibitor of the receptor activator of the nuclear factor kappa-β ligand (RANKL). OPG prevents the binding of RANKL to its membrane-bound receptor-RANK which results in an inhibition of bone resorption by downregulation of osteoclastogenesis [1]. OPG is secreted mainly by osteoblasts but is also released by many organs and cell types such as lung, heart, kidney, liver, skin, bone marrow, vascular muscle cells, lymphocytes and dendritic cells [2]. Serum OPG levels are associated with endothelial dysfunction, coronary calcification and the development of atherosclerosis [3,4]. Elevated OPG concentrations were also observed in individuals with metabolic syndrome, diabetes, hypertension and heart failure [5,6]. OPG concentrations increase with the progression of CKD, reaching the highest level in end-stage renal disease patients [7,8]. Elevated OPG concentrations may predict renal disease in elderly patients [9]. High OPG levels are associated with poorer prognosis of the progression of CKD [10]. The cause of OPG accumulation in ureamic individuals remains unknown. Serum OPG concentrations decrease after successful renal transplantation [11].

CKD is associated with cardiovascular complications such as atherosclerosis, hypertension, heart failure (HF) and coronary artery disease [12]. Left ventricular hypertrophy (LVH), which is an independent predictor of cardiovascular mortality and morbidity, starts in the early stages of CKD [13]. Cardiovascular complications are the main cause of death in CKD patients [14]. OPG is a marker of higher cardiovascular mortality in CKD patients independent of gender, age and other cardiovascular risk factors [15]. High OPG concentrations in CKD individuals correlate with inflammatory markers, endothelial dysfunction and oxidative stress [16].

The purpose of our study was to investigate the association between OPG concentrations and cardiovascular complications such as left ventricle hypertrophy, systolic and diastolic function of the left ventricle and dysfunction of the right ventricle in chronic kidney disease patients not treated with dialysis. We also examined the relation between OPG and the amount of pericardial fluid.

## 2. Methods

### 2.1. Design

An observational study in male patients with chronic kidney disease not treated with dialysis was performed. The inclusion criterion was eGFR lower than 60 mL/min/1.73 m^2^.

### 2.2. Patients

Participants were recruited for the study between November 2018 and February 2020. Patients visited the Nephrological Outpatient Clinic of Military Institute of Medicine, in Warsaw, Poland for a routine checkup. If they fulfilled the inclusion criteria and agreed to participate in the study, a new visit was arranged for further assessment. The inclusion and exclusion criteria are summarized in Table 1. Patients were asked to come for the examination after an overnight fast. Each participant signed an informed consent. One hundred and one men with chronic kidney disease and with eGFR lower than 60 mL/min/1.73 m^2^ were included in the study. The local ethics committee accepted the study protocol (Bioethics Committee in Military Institute of Medicine in Warsaw, Poland, IRB acceptance number 120/WIM/2018 obtained 22 August 2018).

Blood samples were drawn from the fasted patients and were immediately transported to the local Department of Laboratory Diagnostics. Serum creatinine concentrations were measured using the Jaffe method (Gen.2; Roche Diagnostics GmbH, Rish-Rotkreuz, Switzerland). Samples for measuring osteoprotegerin (OPG) and TNF-alpha were kept frozen at −80 °C. OPG concentrations and TNF-alpha were assessed using the Luminex MAGPIX platform.

GFR was evaluated using the short modification of diet in renal disease (MDRD) formula. GFR in mL/min per 1.73 m^2^ = 175 × SerumCr − 1.154 × age − 0.203 × 1.212 (if patient is black) × 0.742 (if female).

Bioimpedance spectroscopy (BIS) was performed using a body composition monitor (Fresenius Medical Care) in supine position after a five-minute rest. Electrodes were placed on one hand and one foot in a tetrapolar configuration.

Echocardiography was performed using a GE Logiq P6 manual ultrasound. Transthoracic echocardiography included two-dimensional and doppler studies according to American Society for Echocardiography guidelines for obtaining images, chamber dimensions and assessment of transvalvular flow [17].

### 2.3. Defining the Cardiovascular Complications

1. Heart failure is classified into 3 phenotypes depending on the left ventricle ejection fraction (EF). Heart failure with EF ≤ 40% is defined as heart failure with reduced ejection fraction—HFrEF. Heart failure with ejection fraction from 41–49% is defined as heart failure with mildly reduced ejection fraction—HFmrEF. When EF is ≥ 50% but there are symptoms of heart failure, it is defined as heart failure with preserved ejection fraction (HFpEF) [18].
2. The value of tricuspid annular plane systolic excursion (TAPSE) is most frequently used to assess the right heart dysfunction. The currently recommended lower limit cut-off for TAPSE is < 17 mm [19].
3. Impaired relaxation is responsible for the development of left ventricle diastolic dysfunction. The mitral valve inflow assessment is the most frequently used technique to evaluate the diastolic function of the left ventricle. The E wave reflects the flow through mitral valve in the early diastolic and the A wave—in the later atrial contraction. An E/A ratio ≥ 1 is the correct value. Impaired relaxation is defined as reversal of the normal E/A ratio (<1) [20]. The impaired relaxation divides into 3 stages, with the reversal of E/A < 1 to E/A > 1 (pseudonormalization) and E/A > 2 (restriction).

Using tissue doppler, the value of E/E’ is measured. E/E’ ratio > 9 is helpful to diagnose the diastolic dysfunction of the left ventricle [21].

The increased size of the left atrium (LA) is another indicator of impaired relaxation. When two-dimensional (2D) echocardiography is used, the systolic surface area of the left atrium is recommended to be less than 20 cm^2^. The correct width of the LA is ≤40 mm. More accurate than the area-length method is the estimation of the left atrium volume index (LAVi) [21]. LAVi > 32 mL/m^2^ is related to higher cardiovascular mortality [21]. The upper normal limit for 2D echocardiographic LA volume is ≤ 34 mL/m^2^ [22].

4. LVH develops with the increase in myocardial thickness. The correct value of end-diastolic interventricular septum thickness (IVS) does not exceed 12 mm [23].

### 2.4. Statistical Analysis

The results are presented as means ± standard deviations (SD) for normally distributed data or medians and ranges or interquartile ranges (IQR) for non-normally distributed variables. The Kolmogorov–Smirnov test was used for evaluating distributions for normality. For correlation analysis, Spearman ρ was applied. Differences between groups were assessed using Student’s *t*-test for normally distributed data and the non-parametric Mann–Whitney test for non-normally distributed parameters. For gradual change estimation across categories, one-way ANOVA with linear trend analysis, Jonckheere–Terpstra test for trend or chi-square test for trend were applied, where appropriate. To control for confounders, logistic regression was used. A *p* value < 0.05 was considered to be statistically significant. Statistical analyses were performed using IBM SPSS v. 25.0 software.

## 3. Results

The study sample consisted of 101 male patients with chronic kidney disease and eGFR lower than 60 mL/min/1.73 m^2^ not treated with dialysis. The median age of the study participants was 66 years (range: 59–72). There were 37 patients in the G3A group (GFR 45–59 mL/min/1.73 m^2^), 32 in the G3B group (GFR 30–44 mL/min/1.73 m^2^) and 32 in the G4–G5 group (GFR <30 mL/min/1.73 m^2^). Median OPG concentration was 425.61 (range 152.89–1239.35 pg/mL). We observed significant negative correlations between OPG concentrations and GFR (ρ = −0.401, *p* < 0.001) and between OPG concentrations and age (ρ = 0.422, *p* < 0.001). OPG concentrations were higher in patients with lower GFR and in elderly individuals. Clinical data and prognostic factors for unfavorable CKD and cardiovascular outcomes are presented in Table 2. 

The differences in OPG concentrations were analyzed and compared with the measurements from echocardiography examinations such as systolic and diastolic function of the left ventricle, the thickness of the interventricular septum, the size of the left atrium, the presence of pericardial fluid and the right ventricle function (Table 3). When appropriate, the results were adjusted for prognostic factors for unfavorable cardiovascular outcomes.

### 3.1. Systolic Function of the Left Ventricle

OPG concentrations were significantly higher in patients with reduced EF (*p* = 0.044). However, there were only 2 patients with reduced EF in our study. 

### 3.2. Left Ventricle Hypertrophy

OPG concentrations were significantly higher in patients with higher myocardial thickness (*p* = 0.006).

This association was independent of the presence of hypertension and BMI in the logistic regression model, where OR_adj_ = 1.004 (*p* = 0.010; 95%CI = (1.001; 1.007); Table 4).

### 3.3. The Size of LA and the Diastolic Dysfunction of the Left Ventricle

OPG concentrations were significantly higher in patients with elevated size of the left atrium (*p* = 0.003). The association with the size of the left atrium was independent of hydration status, hypertension and BMI in the logistic regression model, where OR_adj_ = 1.004 (*p* = 0.045; 95%CI = (1.000;1.007); Table 4). A significant association between OPG and impaired relaxation or elevated E/E’ value was not observed. Nevertheless, median OPG concentration was higher when E/E’ value was >9 than ≤9, although this difference did not reach the statistical significance (*p* = 0.081).

### 3.4. Dysfunction of the Right Ventricle

A significant association between OPG and dysfunction of the right ventricle was not found. However, the systolic function of the right ventricle was normal in almost all study participants.

### 3.5. The Presence of Pericardial Fluid

OPG concentrations were significantly higher in patients with pericardial fluid in the echocardiography examination (*p* = 0.003).

This association was independent (on the border of significance) from the hydration effect in the logistic regression model, where OR_adj_ = 1.004 (*p* = 0.050; 95%CI = (1.000;1.008); Table 4).

### 3.6. Myocardial Infarction in the Past

OPG concentrations were significantly higher in patients with past myocardial infarction (*p* = 0.016).

## 4. Discussion

The role of OPG in the development of cardiovascular disease is not well understood. Many studies report that OPG concentrations rise along with a decrease in glomerular filtration rate [24,25]. Cardiovascular complications are the main cause of mortality and morbidity in CKD [9]. Patients with impaired kidney function, even with mildly decreased GFR, should be considered as being at high risk of the development of cardiovascular disease. Cardiovascular complications in CKD patients include HF, LVH, hypertension, atherosclerosis, metabolic syndrome and coronary artery disease. The results of our study confirm these observations. An inverse correlation between OPG level and GFR (*p* < 0.001) was observed in our study. We also found a significant positive correlation between OPG and age (*p* < 0.001), which was also demonstrated in other studies [26].

The role of OPG in CKD is not well understood. The aim of our study was to assess whether OPG is associated with the development of cardiovascular complications in CKD. Some studies have investigated the role of OPG in the progression of cardiovascular complications in CKD [24,27,28]. Transthoracic non-invasive echocardiography is the most accessible and the most frequently used method to assess the thickness of the left ventricular walls, the mass of the left ventricular muscle, the volume of the LA as well as the systolic and diastolic function of the left ventricle. It also enables the examination of the presence of pericardial fluid. The prevalence of LVH increases at each stage of CKD and is strongly associated with an increase in the risk of mortality and cardiovascular events in this group of patients [29,30]. In our study, OPG concentration, independent of hypertension and BMI, was associated with the thickness of the IVS (*p* = 0.010), which leads to the conclusion that OPG is an independent marker of LVH in the studied population. 

Hypertension, LVH, diabetes and obesity are the conditions which lead to diastolic dysfunction of the left ventricle. The augmented mass of the left ventricle results in the rise of left ventricular end-diastolic pressure (LVEDP). Diastolic dysfunction is very often associated with increased LA size, which is the result of high LVEDP. The increased size of the LA may also be caused by hypertension, obesity, atrial fibrillation, coronary artery disease and annular calcification. Large LA diameter may be a strong predictor for an increased risk of cardiovascular death [31]. Diastolic dysfunction of the left ventricle is associated with increased mortality and hospitalization due to heart failure [32]. Echocardiography is the main tool which assesses the diastolic function of the left ventricle. The echocardiographic variables in the measurement of the diastolic function of the left ventricle are mitral E/A ratio and E/E’ value, as E’ depends on LV relaxation. The impaired relaxation is classified into three stages, with the reversal of E/A < 1 to E/A > 1 (pseudonormalization) and E/A > 2 (restriction). The ratio of E/E’ > 9 is associated with an increased risk of cardiovascular and all-cause mortality [21]. In our study, OPG level was significantly related to the size of the left atrium (*p* = 0.045), independent of overhydration, hypertension and obesity. Although we did not find statistically significant relationships between OPG and impaired relaxation or E/E’, we may conclude that OPG can be a significant marker of the development of left ventricle diastolic dysfunction. This results from the fact that OPG was significantly and independently associated with LVH in our study (*p* = 0.010), and LVH is the condition which leads to diastolic dysfunction of the left ventricle. The increased size of the LA, which was also significantly and independently associated with OPG in our study (*p* = 0.045), is in turn the result of increased LVEDP, the condition which is also related to left ventricle diastolic dysfunction. 

There are studies which also evaluated the association of high OPG concentrations with the development of HF. Elevated OPG levels were associated with increased all-cause mortality in hospitalized patients with HF and with increased HF risk among males in the general population [33,34]. In the Copenhagen City Heart Study, which included nearly 6000 women and men, it was reported that OPG concentrations may be associated with traditional risk factors of atherosclerosis, such as hypertension, diabetes and hypercholesterolemia and with subclinical atherosclerosis and cardiovascular disease [35]. In the Tromsø study which involved 6265 individuals without prior myocardial infarction or ischemic stroke, increased serum OPG level was associated with future risk of myocardial infarction, ischemic stroke and total and cardiovascular mortality [36]. Additionally, OPG level was observed to be significantly higher in patients with acute myocardial infarction and unstable angina in comparison with healthy individuals [37]. ST-elevation myocardial infarction (STEMI) patients with no-reflow phenomenon during primary coronary angioplasty and with the development of left ventricular remodeling after myocardial infarction had higher OPG concentrations [38]. In our study we also analyzed the differences in OPG concentrations between patients with correct EF and with impaired EF and between those who underwent myocardial infarction and those without acute coronary syndrome in the past. We may conclude that in the group of patients with CKD not treated with dialysis, OPG concentrations are significantly higher in individuals with EF < 50% and in those after myocardial infarction in the past in comparison with patients with EF ≥ 50% and those without a medical history of acute coronary syndrome (*p* = 0.044; *p* = 0.016). However, in our study there were only two patients with impaired EF < 50%. Therefore, OPG may be an independent marker of systolic heart failure in the studied population.

As it was shown in the study of *Rymarz* et al., OPG was an independent marker which may identify patients with poor prognostic factors, e.g., overhydration in CKD [39]. Pericarditis is one of the complications of uremia. Echocardiography is the simplest and the most accessible method for evaluating the amount of pericardial fluid. In 1978, *Kleiman* et al. described echocardiography as a way to examine the presence of pericardial fluid in CKD patients [40]. Nowadays, transthoracic echocardiography enables the visualization of the exact amount of pericardial fluid and its sequestration. In our study, we also evaluated the amount of pericardial fluid in CKD patients using transthoracic echocardiography. In our report, OPG is significantly and independently related to overhydration. The presence of pericardial fluid in the studied population is also associated with overhydration. Thus, we can conclude that OPG is an independent marker of the presence of pericardial fluid in the studied population (*p* = 0.050).

The aim of our study was also to evaluate the association between OPG and the dysfunction of the right ventricle in CKD patients. Transthoracic echocardiography is very useful for evaluating the dysfunction of the right ventricle, and the simplest method to do so is to visualize the TAPSE. Dysfunction of the right ventricle is one of the leading predictors of mortality and heart failure in end-stage renal disease patients [41]. The study of *Tamulènaitè* et al. showed that end-stage renal disease patients treated with hemodialysis had more frequently impaired right ventricle function compared with healthy controls [42]. There are fewer studies which examine the function of the right ventricle in CKD in comparison to those which investigate the function of the left ventricle. The geometry of the right ventricle is much more complicated than the left ventricle, therefore, it is more difficult to evaluate the function of the right ventricle. Using the TAPSE as one of the methods to calculate right ventricle dysfunction, we did not find a significant association between OPG and the dysfunction of the right ventricle in the studied population. However, it is worth mentioning that only three patients had a TAPSE < 17 mm and those patients had elevated OPG concentrations. 

The limitation of our study is a relatively small population. An increased number of participants could have enabled us to divide the studied population into subgroups with different GFRs. Another limitation is the fact that we evaluated the size of the LA by measuring the surface area of the LA using two-dimensional transthoracic echocardiography rather than estimating LAVi, which is the most recommended way to measure the size of the LA. It would be more challenging to estimate left ventricle hypertrophy by measuring the thickness of the left ventricle posterior wall.

## 5. Conclusions

Cardiovascular complications, such as atherosclerosis, hypertension, heart failure, coronary artery disease and left ventricular hypertrophy, are the main causes of mortality and morbidity in CKD. Patients with impaired renal function are at high risk of developing of cardiovascular disease. Early detection of cardiovascular complications in this group of patients may be helpful to predict the future risk of cardiovascular events. It may also allow the implementation of a therapeutic method before the development of symptomatic heart failure. Serum concentrations of OPG are elevated in CKD. OPG levels are also increased in patients with metabolic syndrome, diabetes, hypertension and heart failure in the general population. OPG levels in CKD patients not treated with dialysis are significantly associated with the thickness of the interventricular septum, the size of the left atrium and the presence of pericardial fluid. OPG concentrations are higher in patients with systolic dysfunction of the left ventricle and in individuals with incidence of myocardial infarction in the past. Therefore, OPG seems to be worth further investigation as a possible marker of cardiovascular complications, such as systolic and diastolic dysfunction of the left ventricle, left ventricular hypertrophy and the presence of pericardial fluid, in CKD patients. 

## Figures and Tables

**Table 1:** The inclusion and exclusion criteria for the studied population.

Inclusion Criteria	Exclusion Criteria
eGFR < 60 mL/min/1.73 m^2^ Age 18–80 years	eGFR ≥ 60 mL/min/1.73 m^2^ Renal replacement therapy or its requirement within the following 3 months Clinical signs of infection Metal parts in the body Physical exertion the day before the examination Alcohol consumption the day before the examination Lack of agreement to participate in the study

eGFR, estimated glomerular filtration rate.

**Table 2:** Clinical characteristics of the study participants and prognostic factors for unfavorable CKD and cardiovascular outcomes.

	*n*	Total (*n* = 101)	CKD Stage			p_trend_
			3A (*n* = 37)	3B (*n* = 32)	4–5 (*n* = 32)	
OPG [pg/mL], median (IQR)	101	425.61 (298.59–560.39)	335.38 (262.50–429.11)	467.63 (356.21–593.31)	499.71 (342.11–677.41)	**<0.001**
Age, median (IQR)	101	66 (59–71)	67 (57–72)	70 (65–75)	61 (54–68)	0.176
Creatinine [mg/dL] median (IQR)	101	1.9 (1.5–2.8)	1.5 (1.4–1.6)	1.9 (1.8–2.1)	3.4 (2.8–4.9)	**<0.001**
eGFR [mL/min/1.73 m^2^], mean ± SD	101	37 ± 14	51 ± 5	37 ± 4	19 ± 6	**<0.001**
Overweight or obesity, %	98	77.6%	83.8%	74.2%	73.3%	0.296
%fat, mean ± SD	85	30.0 ± 8.4	30.9 ± 7.1	31.5 ± 8.3	27.6 ± 9.8	0.158
%LTM, mean ± SD	85	58.0 ± 11.5	57.4 ± 10.2	56.1 ± 10.7	60.6 ± 13.7	0.329
FTI	85	12.3 ± 4.8	12.6 ± 4.2	13.0 ± 5.1	11.2 ± 5.3	0.298
LTI	85	16.7 ± 2.9	16.7 ± 2.6	16.1 ± 2.8	17.2 ± 3.5	0.624
OH > 1.0 L, %	85	28.2	12.1%	30.8%	46.2%	**0.004**
ECW/ICW ratio, mean ± SD	85	0.84 ± 0.10	0.82 ± 0.09	0.84 ± 0.08	0.85 ± 0.12	0.241
Hypertension, %	98	40.8%	29.7%	35.5%	60.0%	**0.014**
Myocardial infarction in the past, %	101	18.8%	13.5%	18.8%	25.0%	0.226
TNF-alpha [pg/mL], median (IQR)	101	4.37 (3.50–5.52)	4.06 (3.28–4.82)	4.36 (3.39–5.23)	5.32 (4.39–6.88)	**<0.001**

OPG, osteoprotegerin; eGFR, estimated glomerular filtration rate; LTM, lean tissue mass; FTI, fat tissue index; LTI, lean tissue index; OH, overhydration; ECW, extracellular water; ICW, intracellular water; TNF-alpha, tumor necrosis factor alpha; *p*-values < 0.05 are marked in bold.

**Table 3:** OPG concentrations in the groups divided according to EF: the presence of myocardial infarction in the past, the thickness of IVS, the size of LA, relaxation, the E/E’ value, TAPSE and the presence of pericardial fluid.

	*n*	Median	Range	*p*-Value
EF				
≥50%	99	419.90	152.89–1239.35	**0.044**
<50%	2	703.70	660.01–747.38	
Myocardial infarction in the past				
No	82	380.75	152.89–1239.35	**0.016**
Yes	19	490.28	272.70–910.38	
IVS				
≤12 mm	80	361.79	176.02–910.38	**0.006**
>12 mm	21	529.01	152.89–1239.35	
LA				
<20 cm^2^	32	335.38	199.16–902.30	**0.003**
≥20 cm^2^	69	463.34	152.89–1239.35	
Relaxation				
Correct	16	325.27	152.89–687.31	0.154
Impaired: (I, II, III stage)	77	419.90	176.02–1239.35	
E/E’				
≤9	34	355.00	152.89–1239.35	0.081
>9	59	436.39	177.48–910.38	
TAPSE				
<17 mm	3	529.01	250.91–536.07	0.904
≥17 mm	98	422.76	152.89–1239.35	
Pericardial fluid				
No	90	393.27	152.89–910.38	**0.003**
Yes	11	672.29	280.04–1239.35	

EF, ejection fraction; IVS, interventricular septum thickness; LA, left atrium; TAPSE, tricuspid annular plane systolic excursion; *p*-values < 0.05 are marked in bold.

**Table 4:** The risk of selected cardiovascular outcomes depending on OPG concentrations—crude results and adjusted for prognostic factors.

Cardiovascular Outcome	Crude Results		Adjusted Results	
	OR (95% CI)	*p*-Value	OR (95% CI)	*p*-Value
IVS ^a^ > 12 mm	1.004 (1.001–1.006)	0.007	1.004 (1.001–1.007)	**0.010**
LA ^b^ ≥ 20 cm^2^	1.004 (1.001–1.007)	0.009	1.004 (1.0001–1.007)	**0.045**
Pericardial fluid ^c^	1.006 (1.002–1.009)	0.002	1.004 (0.9999–1.008)	0.050

^a^ adjusted for hypertension, BMI; ^b^ adjusted for BMI, overhydration, hypertension; ^c^ adjusted for overhydration; *p*-values < 0.05 are marked in bold.

## Data Availability

The data presented in this study are available on request from the corresponding author. The data are not publicly available due to Polish General Data Protection Regulation.
